# Natural disasters and suicidality in Australia: a systematic review

**DOI:** 10.1007/s00127-026-03160-x

**Published:** 2026-06-26

**Authors:** Mike Trott, C. Lockett, U. Arnautovska, S. Kisely, D. Crompton, D. Siskind

**Affiliations:** 1 Queensland Centre for Mental Health Research, Wacol, Australia; 2 Metro South Addiction and Mental Health Services, Brisbane, Australia; 3 Faculty of Health, Medicine and Behavioural Sciences, University of Queensland, Brisbane, Australia; 4 Griffith University, Brisbane, Australia; 5 Queensland University of Technology, Brisbane, Australia; 6 Princess Alexandra Hospital, Ground Floor, Building 33, Brisbane, Australia

**Keywords:** Suicide, Natural disaster, Flood, Drought, Fire

## Abstract

**Purpose:**

To systematically review Australian evidence examining associations between exposure to natural disasters and suicidal behaviours, including suicidal ideation, suicide attempts, and suicide mortality.

**Methods:**

A systematic review was conducted in accordance with a pre-registered protocol (CRD42023471489), PRISMA, and SwiMS guidelines. Databases were searched in May 2026. Eligible studies were Australian observational studies examining droughts, floods, bushfires, heatwaves, or storms in relation to suicidal ideation, suicide attempts, or death by suicide. Risk of bias was assessed using Joanna Briggs Institute tools. Findings were narratively synthesised.

**Results:**

Twelve studies met inclusion criteria. Evidence linking natural disasters to suicide mortality was mixed and inconsistent, particularly in short-term post-disaster analyses. In contrast, individual-level studies more consistently reported increased odds of suicidal ideation and self-harm following disaster exposure. Associations were strongest for prolonged drought, heat exposure, displacement, and cumulative or repeated disaster exposure, with several studies demonstrating dose response patterns. Most studies were judged to have moderate risk of bias, commonly due to exposure misclassification and limited control of confounding variables.

**Conclusion:**

In Australia, natural disasters are more consistently associated with non-fatal suicidal behaviours than with suicide mortality. Post-disaster mental health responses should incorporate monitoring of these behaviours beyond the immediate recovery phase.

**Supplementary Information:**

The online version contains supplementary material available at https://doi.org/10.1007/s00127-026-03160-x.

## Introduction

Exposure to natural disasters can have detrimental effects on individuals and communities, with suicidal behaviours representing one of the most severe and consequential of them. Suicidality exists along a continuum, ranging from suicidal ideation to suicide attempts and death by suicide, each of which may be influenced by distinct mechanisms and risk factors [1, 2]. In Australia, suicide remains a leading cause of death among working-age adults, particularly men [3], and rates are consistently higher in rural and remote areas [4–6], which are also more likely to experience severe environmental stressors such as drought and bushfires [3, 7]. Understanding whether, how, and for whom natural disasters influence suicidality is therefore a critical public health priority in Australia.

Natural disasters can be defined as disasters resulting from naturally occurring hazards, such as earthquakes, storms (such as cyclones and hurricanes), tsunamis, floods, wildfires, and droughts that result in significant challenges to local societies, often leaving devastating impacts on respective communities [8], and are a major and growing public health concern in Australia. Events such as prolonged droughts, catastrophic bushfires, floods, cyclones, and heatwaves have increased in frequency and intensity over recent decades [9], driven in part by climate change and environmental degradation. These events disproportionately affect rural and regional communities, Aboriginal and Torres Strait Islander people, and socioeconomically disadvantaged populations, amplifying existing health inequities [10, 11]. While the immediate impacts of natural disasters are often measured in terms of physical injury, mortality, and infrastructure damage, there is increasing recognition of mental health consequences of natural disasters.

International systematic reviews and meta-analyses have reported that suicidal ideation, attempts, and suicide deaths may increase following natural disasters, particularly among those with pre-existing mental health conditions [12, 13]. However, much of this literature is dominated by studies of sudden impact disasters such as earthquakes and tsunamis in non-Australian settings, with substantial heterogeneity in disaster type, outcome definition, follow-up period, and analytical approach. It is possible that the effects may be different to disasters of slower onset (such as droughts) that are more characteristic of Australia [14]. As a result, the applicability of these findings to the Australian context is uncertain. Primary studies examining disasters and suicidality in the Australian context have produced mixed findings. For example, some analyses report increases in suicidal ideation post bushfire exposure [15], while others report null or inverse associations [16]. To date, however, these studies have not been systematically aggregated.

The aim of this systematic review, therefore, is to synthesise evidence examining associations between exposure to natural disasters and suicidal behaviours in Australian populations.

## Methods

This study was conducted using a pre-published protocol (PROSPERO ID: CRD42023471489), according to the Preferred Reporting Items for Systematic reviews and Meta-Analyses (PRISMA) statement [17] and reported using the Synthesis without meta-analysis (SWiM) in systematic reviews guidelines [18].

### Searches

Searches were conducted on 18th May 2026 on PubMed, PsycINFO, Embase, Web of Science, Cinahl, and Scopus databases, using search terms synonymous with natural disasters and suicidal ideation, suicide attempt, and death by suicide. Full details on the search terms can be found in Supplementary Table 1. The inclusion criteria followed the PICOS framework, and included the following:

Population: Any participants, of any age, in Australia.

Intervention/exposure: Exposure to natural disasters, defined as severe storms (cyclones, tornados), fires (bushfires), droughts, and floods (including tsunamis).

Comparators: No comparators were required for studies to be included.

Outcome: Suicidal ideation (thoughts of ending one’s life, regardless of intent or plan), suicide attempt (non-fatal self-directed injurious behaviour with intent to die), including self-harm (self-directed injurious behaviour that may or may not be suicidal in intent), and/or death by suicide. Where included studies assessed self-harm without specifying suicidal intent or used composite measures, findings were reported accordingly.

Study types: All kinds of peer-reviewed observational study were included. Furthermore, the reference lists of review studies were screened.

### Screening and data extraction

Duplicates were removed automatically using Covidence software. Title and abstracts of eligible studies were independently screened by two reviewers (MT; CL), as were full texts of articles. A third reviewer (UA) was available to arbitrate any conflicts (this was not needed). Following full text review, data were extracted onto a spreadsheet by two independent reviewers (MT; CL, with UA checking both sets of data extraction for accuracy), based on recommendations from the Joanna Briggs Institute (JBI) guidelines for systematic reviews [19].

### Risk of bias

Risk of bias was assessed by MT and CL independently using the Joanna Briggs Institute (JBI) tool that aligns with the respective component study design. UA made a final decision when there were conflicts.

### Data synthesis

Studies meeting inclusion criteria were narratively synthesised in accordance with SWiM guidelines [18]. Considering substantial heterogeneity in study design, disaster type, outcome definition, and analytical approach, meta-analysis was not appropriate. Studies were grouped by primary disaster type (bushfire, flood, drought, heatwave, and cumulative/unspecified disaster exposure). Within each group, the direction of effect for each outcome (suicidal ideation, suicide attempt, self-harm, suicide mortality) was assessed descriptively. Risk-of-bias ratings informed interpretation, and conclusions were drawn more cautiously from ecological and case-series designs than from individual-level longitudinal studies. Where studies reported inconsistent findings within a disaster type, these inconsistencies are explicitly noted and plausible explanations discussed.

## Results

**Fig. 1:**
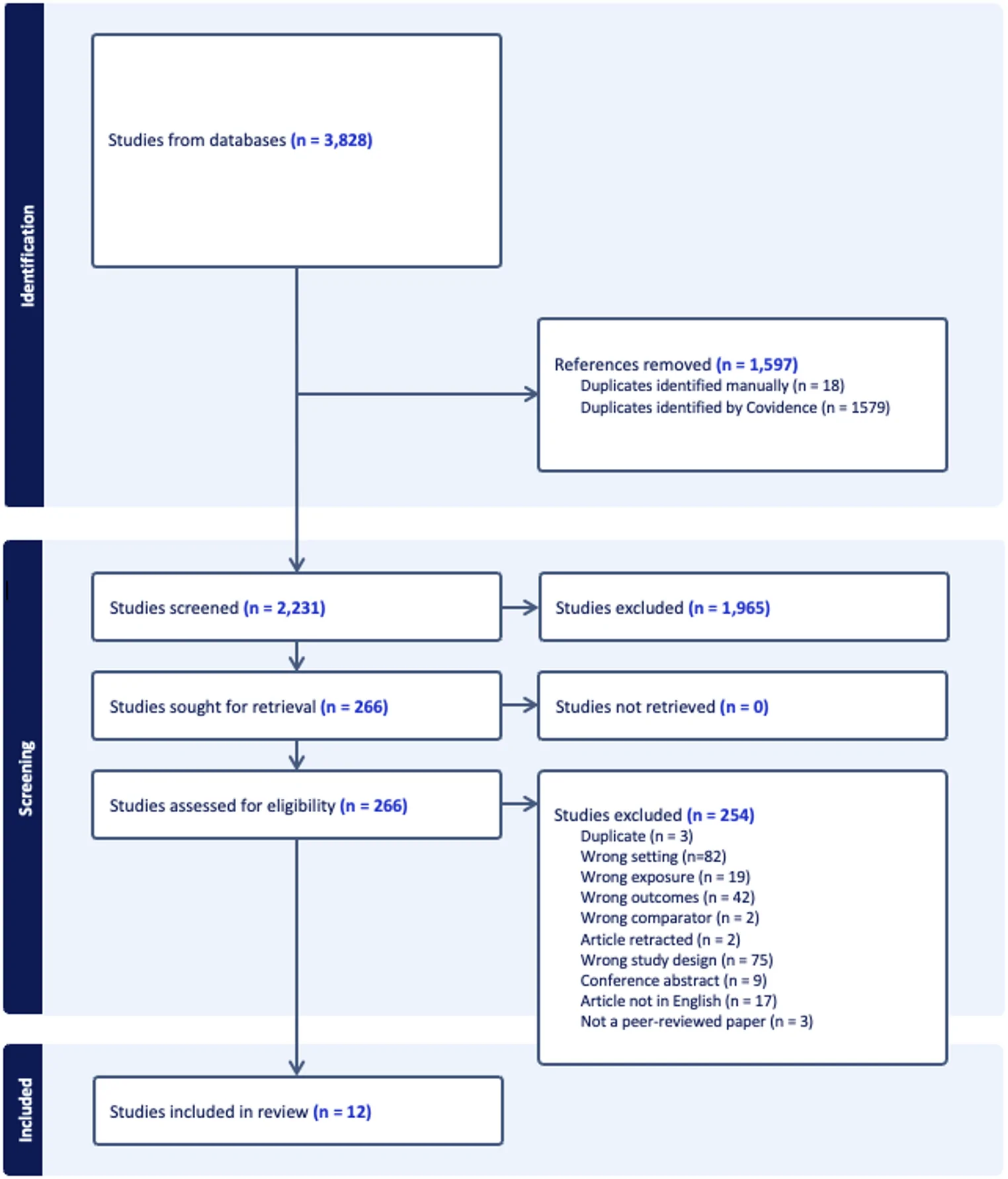
PRISMA flowchart showing included studies

A total of 3,828 initial studies were identified, of which 1,597 studies were removed as duplicates, with remaining 2,231 studies included in the title and abstract review. Of these, 266 studies were deemed eligible for full-text review. After full-text review, 12 studies were included (see Fig. 1), of which six studies examined droughts, three studies floods, three studies bushfires, and one study respectively examined heatwaves and unspecified ‘natural disasters’ (some studies examined more than one disaster type). Out of 12 included studies, six studies examined suicidal ideation, two studies examined suicide attempts, and five studies examined death by suicide. Note that du Toit et al. [20] and Beames et al. [15] were drawn from the same longitudinal cohort, and report outcomes at different timepoints; findings from these two studies were therefore reported as separate studies but interpreted together. Table 1 shows full descriptive information for included studies. Regarding study design, there were two longitudinal cohort studies, four cross-sectional, four ecological or quasi-experimental time-series, and two case series. Most studies were judged as having a moderate risk of bias according to the JBI tools (see Supplementary Table 2), with limitations in exposure ascertainment (frequently self-reported), and incomplete control of confounding variables in the cross-sectional and time-series analyses. Detailed information regarding outcomes of each study can be found in Table 2.

**Table 1:** Descriptive characteristics of included studies

Author(s)	Study type	Type of natural disaster	Location	Suicide/suicide attempt/ideation	*n*	Mean age (SD)	Percentage female	Follow up time	Risk of bias
Beames et al. [15]	Cross-sectional	Bushfire	New South Wales	Ideation	5,866	NR	49.8	NR	Low
Cowlishaw et al. [21]	Cross-sectional	Bushfire	Victoria	Ideation	736	NR	61.5	5 years	Moderate
De Leo et al. [16]	Ecological	Flood	Queensland	Death by suicide	Various	NA	NA	6 months	Moderate
Dey et al. [28]	Time-series/quasi-experimental	Heatwaves	New South Wales	Ideation	132,296	NR (adolescents between 11–17)	64.4	7 years	Moderate
du Toit et al. [20]	Longitudinal	Bushfire	New South Wales	Ideation	2,967	13.8 (0.5)	52.0	2 years	Low
Edwards et al. [23]	Longitudinal	Fires/floods and drought	National	Ideation and attempts	2,908	NR (adolescents)	NR	7 years	Low
Guiney et al. [26]	Retrospective	Drought	Victoria	Death by suicide	3,523	NR	NR	7 years	Moderate
Hanigan et al. [24]	Ecological	Drought	New South Wales	Death by suicide	NR	NR (adults aged between 30–49 years)	NR	38 years	Moderate
Kunde et al. [27]	Cross-sectional	Drought	Queensland, New South Wales	Death by suicide	18	53 (13.4)	0	NR	Moderate
Matthews et al. [22]	Cross-sectional	Floods	New South Wales	Ideation	2,180	NR	69	6 months	Moderate
Reifels et al. [29]	Cross-sectional	Unspecified (denoted as “natural disasters” only)	National	Ideation and attempt	8,841	NR	54.4	NR	Low
Xu et al. [25]	Ecological	Drought	Murray–Darling Basin (NSW, Victoria, Australian Capital Territory, Queensland, South Australia)	Death by suicide	50,543	NR	NR	10 years	Moderate

**Table 2:** Outcomes

Author(s)	Exposure	Outcome	Effect measure	Effect estimate (95% CI)	Adjustment variables
Beames et al. [15]	Bushfire (personal harm)	Ideation	OR	1.95 (1.27–3.00)	Adverse childhood experiences; Mental illness history; Gender; Sexual orientation; Perceived household wealth; Language spoken at home; COVID-19 diagnosis/quarantine
Cowlishaw et al. [21]	Anger problems in a bushfire-affected population	Ideation	OR	4.18 (1.73–10.09)	Gender; age; employment status; probable posttraumatic stress disorder
du Toit et al. [20]	Bushfire harm (evacuated, property damage, or injury)	Ideation	OR	0.88 (0.29–2.14)	Baseline suicidality; gender identity; sexuality; mental health diagnosis; adverse childhood experiences; COVID diagnosis/quarantine
Guiney et al. [26]	Drought period (Victorian drought years, 2001–2007)	Death by suicide	NR	NR – Descriptive analysis reports no consistent increase in farming suicides during drought years	None reported
Hanigan et al. [24]	Drought severity	Death by suicide	RR (rural males)	15% (8–22%) risk increase when drought index rose from 1 st to 3rd quartile	Season; age; region
			Change in annual suicide count	−0.72 suicides/year (− 1.32 to − 0.01)	Season; age; region
Kunde et al. [27]	Drought as a contextual farming stressor	Death by suicide	Descriptive	Drought was identified as a common contributing factor across suicide pathways (no effect estimates reported)	None reported
Xu et al. [25]	Moderate drought duration (months in past 12 months)	Death by suicide	Poisson regression	2% increase in suicide rate per additional month of moderate drought (no 95% reported)	year; season; proportion Indigenous population; unemployment; household income; proportion farmers; proportion health professionals; education
	Extreme drought duration (months in past 12 months)			32% increase in suicide rate per additional month of extreme drought (no 95% reported)	
	Average monthly maximum temperature (+ 1 °C)			8% increase in suicide rate per 1 °C increase (no 95% reported)	
De Leo et al. [16]	Flood exposure	Death by suicide	Poisson regression comparing suicide rates pre-vs post flood	No significant increase in suicide rates during the 6 months following the floods (no effect estimates reported)	None reported
Dey et al. [28]	Heatwave	Ideation	Percent change in rate of ED presentations for suicidal ideation	For every 1 °C increase above the average daily mean temperature, ED presentations increased by 1.3% (95% NR)	Day-of-week; school term; climate zone
Matthews et al. [22]	Floods: where home of significant other was affected	Ideation	OR	1.37 (0.56–3.36)	Age; sex; Aboriginal and Torres Strait Islander status; relationship status; education qualification; employment status; income support
	Floods: where suburb was affected		OR	1.46 (0.60–3.56)	
	Floods: where non-liveable areas affected		OR	1.75 (0.71–4.32)	
	Floods (where liveable areas affected)		OR	2.59 (1.02–6.62)	
	Floods: where home was evacuated		OR	2.58 (0.97–6.88)	
	Floods: displaced < 6 months		OR	2.46 (0.90–6.75)	
	Floods: displaced > 6 months		OR	3.17 (0.93–10.39)	
	Floods: where business/farm affected		OR	2.88 (1.06–7.85)	
	Floods: where business was evacuated		OR	2.94 (1.03–8.40)	
	Floods: cumulative exposure – any 1 of the above		OR	0.79 (0.28–2.26)	
	Floods: cumulative exposure – any 2 of the above		OR	0.56 (0.19–1.61)	
	Floods: cumulative exposure – any 3 of the above		OR	1.74 (0.68–4.46)	
	Floods: cumulative exposure – any 4 of the above		OR	1.93 (0.74–5.03)	
	Floods: cumulative exposure – any 5 of the above		OR	7.87 (2.48–25.05)	
Edwards et al. [23]	Fire/flood: self-reported exposure in previous 12 months	Suicide attempt	OR	1.44 (0.84–2.49)	Child age; child sex; child is a migrant; child is Aboriginal or Torres Strait Islander; moved house; regional area; state; SEIFA Index of Advantage and Disadvantage
		Suicidal ideation	OR	1.61 (1.07–2.42)	
	Fire/flood: consecutive exposure across time	Suicide attempt	OR	1.09 (0.78–1.51)	
		Suicidal ideation	OR	1.13 (0.89–1.47)	
	Drought: self-reported exposure in previous 12 months	Suicide attempt	OR	1.60 (0.90–2.83)	
		Suicidal ideation	OR	0.99 (0.63–1.58)	
	Drought: consecutive exposure across waves	Suicide attempt	OR	0.87 (0.62–1.23)	
		Suicidal ideation	OR	0.70 (0.53–0.93)	
	Multiple disaster exposure: cumulative consecutive exposure across disaster types	Suicide attempt	OR	1.10 (0.93–1.29)	
		Suicidal ideation	OR	0.99 (0.87–1.12)	
Reifels et al. [29]	Natural disaster: any lifetime exposure	Suicidal ideation	OR	1.15 (0.82–1.60)	Age; gender; country of birth; marital status; qualification; labor force status; relative socioeconomic disadvantage; household location; man-made disaster exposure; exposure to any other traumatic event
		Suicide attempt	OR	1.26 (0.63–2.54)	
	Natural disaster: one lifetime exposure	Suicidal ideation	OR	1.12 (0.73–1.71)	
		Suicide attempt	OR	0.75 (0.27–2.04)	
	Natural disaster: two or more lifetime exposures	Suicidal ideation	OR	1.26 (0.76–2.09)	
		Suicide attempt	OR	2.21 (1.04–4.71)	

OR=odds ratio; RR-relative risk; NR = not reported; SEIFA = Socio-Economic Indexes for Areas

### Bushfires

In adolescents (aged 16–25 years; *n =* 5,866), Beames et al. [15] examined exposure to bushfire-related harm, and reported that bushfire exposure was associated with increased odds of suicidal ideation. They also reported additive effects when bushfire exposure co-occurred with other disasters. In a subsequent longitudinal analysis of the same cohort as used in Beames et al. [15], du Toit et al. examined mental health outcomes at 24-month follow-up among adolescents who had reported bushfire harm (*n* = 2,967; 167 bushfire-harmed). Contrary to Beames et al., bushfire harm was not a significant predictor of suicidality symptoms at 24 months [20]. In adults, Cowlishaw et al. [21] assessed mental health outcomes across several years after a major bushfire event in Victoria (*n* = 736). They reported that significant anger problems within a bushfire-affected adult population were associated with suicidal ideation.

### Floods

Matthews et al. [22] conducted a cross-sectional study (*n* = 2,180) six months after river flooding in Northern New South Wales. Compared with individuals reporting no flood exposure within the same region, those whose homes were inundated reported significantly increased odds of suicidal ideation, with adjusted odds ratios ranging from 2 to 3 depending on exposure severity. There was a dose–response relationship where the odds of suicidal ideation increased with the number of flood-related exposures (e.g. suburb damage, home or business inundation, evacuation, displacement). Furthermore, people who remained displaced six months post-flood exhibited elevated risks of psychological morbidity (e.g. PTSD), with suicidal ideation showing the same directional pattern. In contrast, De Leo et al. [16] examined suicide mortality in Ipswich and Toowoomba following the 2011 Queensland floods using linked death data. Comparing suicides in the six months post-flood with the same calendar months across the preceding 11 years, they found no statistically significant increase in suicide rates. In a nationally representative adolescent cohort (aged 14 to 19 years; *n* = 2,908), Edwards et al. [23] reported that exposure to fire or flood in the previous 12 months was associated with increased odds of self-reported self-harm and suicidal ideation compared to those who had not reported being exposed to fire or flood across the previous 12 months, with odds ratios ranging from 1.3 to 1.6. Similarly, Beames et al. [15] included flood exposure within their composite measure of natural disaster exposure among adolescents, and reported increased odds of suicidal ideation among disaster-exposed youth.

### Droughts

Drought exposure was examined primarily in ecological and time-series analyses focusing on suicide mortality. Hanigan et al. [24] analysed suicide mortality in New South Wales from 1970 to 2007 in relation to drought severity, reporting that during drought periods, suicide rates among men aged 30–49 years increased by approximately 15–20% compared with non-drought periods (no sample sizes were reported). These increases were strongest in rural areas and among populations more directly dependent on agricultural conditions. Similarly, Xu et al. [25] examined suicide mortality across the Murray–Darling Basin (*n* = 50,543) and reported that worsening drought conditions were associated with increased suicide risk, with incidence rate ratios ranging from 1.1 to 1.2 per unit increase in drought or temperature indices, with effect sizes being consistent across geography. Descriptive and qualitative evidence from farming-focused studies supported the association between droughts and suicide risk. For example, Guiney et al. [26] conducted a retrospective analysis of farmer suicides during the Millennium Drought in Victoria (*n* = 3,523). Descriptive patterns showed fluctuations in farming suicide counts across the drought period, but no formal statistical test was conducted and no consistent increase was therefore demonstrated. Kunde et al. [27], using a life-chart analysis based on next-of-kin interviews, cumulative drought exposure, financial stress, and erosion of occupational identity were identified as recurring contextual factors in suicide pathways, however this was conducted with a small sample (*n* = 18).

### Heatwaves

Heat exposure was examined in one quasi-experimental time-series study. Dey et al. [28] analysed emergency department presentations for suicidality among young people aged 12–24 years (*n* = 132,296) in New South Wales in relation to ambient temperature and heatwaves, reporting that heatwave days were associated with increased presentations for suicidal ideation and self-harm, with rate increases between 5% and 10%.

### Cumulative and repeated disaster exposure

Several studies examined cumulative or repeated exposure to disasters. For example, Reifels et al. [29], in their nationally representative study (*n* = 8,841), reported that exposure to two or more natural disasters was associated with over two-fold increase in the odds of lifetime suicide attempts, while a single natural disaster exposure was not significantly associated with the incidence of suicide attempts. In adolescents, Edwards et al. [23] found that ‘compound disasters’ (defined as two or more disasters occurring within 12 months) and ‘cascading disasters’ (e.g. drought followed by fire or flood) were associated with increased odds of self-harm and suicidal ideation. In contrast, recurrent consecutive drought exposure was not associated with increased suicidal ideation and, in some models, was associated with lower odds.

## Discussion

This review synthesised the Australian evidence examining associations between natural disasters and suicidal behaviour, including suicidal ideation, attempts, and suicide mortality. As expected, the majority of studies concerned disasters of slower onset such as flooding or drought. Overall, the findings indicate heterogeneous and often inconsistent associations for suicide mortality, contrasted with more consistent evidence for elevated suicidal ideation and self-harm following disaster exposure, particularly for prolonged or cumulative stressors such as drought, heat, and repeated exposure. These patterns align closely with the most recent international literature [12], while also highlighting the relative scarcity of robust, Australia-specific studies.

Our findings extend an updated international systematic review by Zortea and colleagues, who also concluded that evidence linking natural disasters to suicide mortality remains inconsistent, with clearer signals observed for non-fatal outcomes such as suicidal ideation and self-harm [12]. Furthermore, they reported that drought and heatwaves were more frequently associated with increases in suicide deaths, whereas floods and cyclones more commonly showed null or mixed effects on mortality. The Australian evidence extends this pattern with an additional six Australian studies (possibly due to a more specific search strategy), with short-term post-flood analyses of suicide mortality showing no clear increases, while individual-level and cohort studies demonstrating elevated risks of suicidal ideation following flood exposure. Similarly, Kõlves et al. have stated that much of the current evidence linking climate-related exposures to suicidality is ecological, methodologically heterogeneous, and vulnerable to bias, particularly exposure misclassification and residual confounding [30], with these limitations clearly reflected in the Australian literature. The relative rarity of suicide deaths compared with non-fatal suicidal behaviours may also reduce statistical power to detect short-term changes in mortality following disaster events.

Thematic reviews further contextualise these findings. Kemel et al., for example, in their systematic review of bushfire exposure and adolescent mental health, reported consistent associations between bushfire exposure and adverse mental health outcomes, including suicidal ideation, particularly where exposure involved perceived life threat, property loss, or ongoing disruption [31]. This pattern, however, was not replicated in the only longitudinal cohort study in this review, with du Toit et al. reporting no significant effect of bushfire harm on suicidality at 24 months, with the authors noting possible resilience or recovery trajectories in this population. This underscores the importance of follow-up timing and individual vulnerability factors (particularly prior mental illness and sexuality diversity) in determining longer-term outcomes, and cautions against assuming that cross-sectional associations necessarily persist over time.

Although suicidality was not the primary outcome in many included studies, the direction of effects aligns with the broader Australian evidence suggesting greater vulnerability among younger populations following disaster exposure. Importantly, Kemel et al. also highlighted that symptoms may persist or worsen over time, supporting concerns that short follow-up windows may underestimate longer-term risk [31]. Broader climate change mental health syntheses also reinforce the findings of the current review. For example, Allison et al. argue that people with severe mental illness may be particularly vulnerable to heat related stressors due to pre-existing psychosocial disadvantage, disrupted care pathways, and heightened sensitivity to environmental stress [32]. However, this population remains underrepresented in disaster–suicide research in the Australian context, representing a critical gap in the current evidence base. Similarly, Aboriginal and Torres Strait Islander peoples are notably absent from the literature as a distinct analytical population, with only Matthews et al. reporting subgroup analyses for this group, and only in relation to flood exposure rather than suicidal outcomes [22]. This represents a critical evidence gap. Aboriginal and Torres Strait Islander peoples experience suicide rates substantially higher than the non-Indigenous population [4–6], and are disproportionately exposed to natural disasters due to the intersection of geographic vulnerability, socioeconomic disadvantage, and historical and ongoing dispossession. Whether disaster exposure further amplifies an already elevated suicide risk in this population remains unknown and warrants urgent investigation. A further conceptual distinction is that the evidence reviewed here does not support a single, unified disaster-suicidality relationship. Rather, the relationship between natural disasters and suicidal outcomes varies by disaster type, exposure intensity, outcome measured, population, and follow-up period. Drought, heatwaves, floods, and bushfires operate through distinct temporal, socioeconomic, and psychological mechanisms and should not be treated as interchangeable exposures. Droughts are slow-onset, chronic stressors that accumulate over years and disproportionately affect agricultural communities through financial strain and occupational identity erosion [12, 24]. Floods, on the other hand, are sudden-onset events that may cause acute trauma, displacement, and property loss, but can also trigger short-term community cohesion effects that temporarily attenuate distress. Heatwaves operate through largely physiological and neurobiological pathways with acute temporal signatures. Bushfires may combine acute trauma with prolonged recovery and displacement. The Australian literature included in this review did not show a clear divergence by disaster onset type, with both rapid- and slow-onset exposures more consistently associated with non-fatal outcomes such as suicidal ideation than with suicide mortality.

Several factors likely explain the heterogeneity observed across Australian studies. First, exposure measurement is imprecise, with many studies relying on area-level classifications that may not reflect individual experience or severity of individual exposure. Second, temporal dynamics are rarely captured; for example acute disasters may be followed by short-term community cohesion (“honeymoon effects”) [30, 33], while distress related to financial strain, displacement, service disruption, and recovery fatigue may emerge months or years later. Third, statistical power is often limited, particularly for suicide mortality outcomes, which are rare events and sensitive to year-to-year variation [22]. The divergence between suicide mortality and non-fatal outcomes is also consistent with international evidence suggesting that suicidal ideation may be more sensitive to psychosocial disruption than suicide deaths, and may therefore serve as earlier indicators of disaster-related mental health risk [29].

## Directions for future work

From a public health perspective, the clearest implication of the Australian evidence is a need to incorporate suicide ideation screening and, if indicated, a more comprehensive suicidal risk assessment into post-disaster mental health responses, particularly beyond the immediate response. The consistency of findings for non-fatal outcomes supports early intervention strategies targeting psychological distress, displacement-related stressors, and cumulative exposure. From a research perspective, priorities should include improving exposure ascertainment, extending follow-up durations, and adopting stronger causal designs, such as time-series analyses or longitudinal cohort studies with repeated measures. Considering projections of increasing disaster frequency and compounding exposures under climate change, studies explicitly modelling cumulative and repeated exposure are urgently needed. Notably absent from this review were studies focusing on priority populations, including Aboriginal and Torres Strait Islander communities, rural and remote populations, disaster responders, and people with severe mental illness. Considering that recent Australian work regarding drought resilience and mental health highlighted the importance of these factors [34], future work should prioritise these locations and populations. Longitudinal studies should also incorporate measurement of contextual stressors that plausibly mediate the disaster-suicidality relationship, particularly potential stressors such as displacement from home, livelihood loss, and financial stress. Furthermore, complementing quantitative designs with qualitative research would further advance understanding of the mechanisms through which disaster exposure elevates suicide risk, including the subjective experience of loss, recovery fatigue, and help-seeking barriers in affected communities.

A key strength of this review is its focus on disasters that are more typical in Australia, such as droughts and floods, as opposed to earthquakes and tsunamis, the results of which may be generalisable to other jurisdictions with similar climatic, geographic, and health system settings. However, the evidence base is limited by heterogeneity in study design, exposure definition, and outcome measurement, with most studies assessed as having moderate risk of bias. Most included studies were rated as moderate risk of bias, primarily due to imprecise exposure ascertainment and incomplete confounding control. Although ecological and case-series designs provide important descriptive and contextual evidence, they cannot establish individual-level causal effects. Conclusions regarding suicide mortality in particular therefore should be interpreted with caution given the small number of mortality studies, their ecological designs, and the inherently limited statistical power for this rare outcome. A further limitation is the heterogeneity of exposure measurement across included studies. As a result of disaster exposure being operationalised in different ways, these constructs reflect meaningfully different dimensions of disaster experience and likely capture different causal pathways to suicidal outcomes. This heterogeneity in exposure measurement likely contributes to the inconsistency in findings across studies and complicates direct comparisons. Lastly, our results could also be subject to publication bias, as only peer-reviewed studies were included; grey literature, government reports, and coronial data were excluded, which may have led to underestimation of the true volume of evidence in this area.

## Conclusions

In Australia, evidence linking natural disasters to suicide mortality remains inconsistent, while associations with suicidal ideation and self-harm are more frequently observed, particularly following prolonged or cumulative exposures to natural disasters. Although these findings are consistent with international reviews, it also highlights substantial gaps in the Australian literature. The available evidence does not support a single, uniform relationship between natural disasters and suicidality; associations vary substantially by disaster type, outcome, exposure intensity, population, and follow-up period, underscoring the need for disaster-specific rather than generic prevention responses.

## Supplementary Information

Below is the link to the electronic supplementary material.

Supplementary Material 1

## Data Availability

No datasets were generated or analysed during the current study.
