# Supplementary material for: Natural disasters and suicidality in Australia: a systematic review
Source: Soc Psychiatry Psychiatr Epidemiol. 2026 Jun 26;61(11):2281–92. doi: 10.1007/s00127-026-03160-x (PMC13694565; doi:10.1007/s00127-026-03160-x)
Supplement: Supplementary file 1 [file 127_2026_3160_MOESM1_ESM.docx]

**Supplementary Table 1**: Systematic review search terms

| Database | Search terms | Number of results |
| --- | --- | --- |
| Scopus | TITLE-ABS-KEY ( ( suicide OR "suicidal ideation" OR "suicide attempt" OR "suicide death" ) AND ( "natural disaster" OR "natural disasters" OR disaster* OR "natural emergency" OR "natural emergencies" OR earthquake OR storm OR flood*OR drought OR cyclone OR tsunami OR wildfire* OR bushfire* OR heatwave OR "heat wave" OR "Black Summer" OR thunderstorm OR hailstorm OR landslide ) | 118 |
| PubMed | ("Suicidal Ideation"[MeSH] OR "Suicide, Attempted"[MeSH] OR "Suicide"[MeSH] OR suicid*[tiab] OR "suicide attempt"[tiab] OR "suicide death"[tiab])  AND  ("Natural Disasters"[MeSH] OR "Floods"[MeSH] OR "Droughts"[MeSH] OR "Fires"[MeSH] OR "Cyclonic Storms"[MeSH] OR "Heat Stress Disorders"[MeSH] OR disaster*[tiab] OR "natural disaster"[tiab] OR earthquake[tiab] OR storm[tiab] OR flood*[tiab] OR drought[tiab] OR cyclone[tiab] OR tsunami[tiab] OR wildfire*[tiab] OR bushfire*[tiab] OR heatwave[tiab] OR "heat wave"[tiab] OR "Black Summer"[tiab] OR thunderstorm[tiab] OR hailstorm[tiab] OR landslide[tiab]) | 818 |
| PsycINFO (through EBSCOHost) | (DE "Suicidal Ideation" OR DE "Attempted Suicide" OR DE "Suicide" OR TI suicid* OR AB suicid*) AND (DE "Natural Disasters" OR DE "Floods" OR DE "Droughts" OR DE "Fires" OR TI disaster* OR AB disaster* OR TI earthquake OR AB earthquake OR TI flood* OR AB flood* OR TI drought OR AB drought OR TI cyclone OR AB cyclone OR TI tsunami OR AB tsunami OR TI wildfire* OR AB wildfire* OR TI bushfire* OR AB bushfire* OR TI heatwave OR AB heatwave OR TI "heat wave" OR AB "heat wave" OR TI "Black Summer" OR AB "Black Summer" OR TI thunderstorm OR AB thunderstorm OR TI hailstorm OR AB hailstorm OR TI landslide OR AB landslide) | 540 |
| Embase | ('suicidal ideation'/exp OR 'suicide attempt'/exp OR 'suicide'/exp OR suicid*:ab,ti OR 'suicide attempt':ab,ti OR 'suicide death':ab,ti) AND ('natural disaster'/exp OR 'flood'/exp OR 'drought'/exp OR 'wildfire'/exp OR 'cyclone'/exp OR 'heat stroke'/exp OR disaster*:ab,ti OR earthquake:ab,ti OR storm:ab,ti OR flood*:ab,ti OR drought:ab,ti OR cyclone:ab,ti OR tsunami:ab,ti OR wildfire*:ab,ti OR bushfire*:ab,ti OR heatwave:ab,ti OR 'heat wave':ab,ti OR 'Black Summer':ab,ti OR thunderstorm:ab,ti OR hailstorm:ab,ti OR landslide:ab,ti) | 1037 |
| Web of Science | ((suicide OR "suicidal ideation" OR "suicide attempt" OR "suicide death") AND ("natural disaster" OR "natural disasters" OR disaster* OR earthquake OR storm OR flood* OR drought OR cyclone OR tsunami OR wildfire* OR bushfire* OR heatwave OR "heat wave" OR "Black Summer" OR thunderstorm OR hailstorm OR landslide)) | 981 |
| Cinahl (through EBSCOHost) | (MH "Suicidal Ideation" OR MH "Suicide, Attempted" OR MH "Suicide" OR TI suicid* OR AB suicid*) AND (MH "Disasters" OR MH "Floods" OR MH "Drought" OR MH "Fires" OR TI disaster* OR AB disaster* OR TI earthquake OR AB earthquake OR TI flood* OR AB flood* OR TI drought OR AB drought OR TI cyclone OR AB cyclone OR TI tsunami OR AB tsunami OR TI wildfire* OR AB wildfire* OR TI bushfire* OR AB bushfire* OR TI heatwave OR AB heatwave OR TI "heat wave" OR AB "heat wave" OR TI "Black Summer" OR AB "Black Summer" OR TI thunderstorm OR AB thunderstorm OR TI hailstorm OR AB hailstorm OR TI landslide OR AB landslide) | 334 |

**Supplementary Table 2**: JBI checklist scoring for each component study

| Study | JBI tool used | Tool question | Rating |
| --- | --- | --- | --- |
| Beames et al. (2023) | Analytical cross-sectional | Inclusion criteria clearly defined | Yes |
|  |  | Study subjects and setting described | Yes |
|  |  | Exposure measured validly/reliably | Yes |
|  |  | Objective, standard outcome criteria used | Yes |
|  |  | Confounding factors identified | Yes |
|  |  | Strategies to deal with confounding | Yes |
|  |  | Outcomes measured validly/reliably | Yes |
|  |  | Appropriate statistical analysis | Yes |
|  |  | Overall risk of bias | Low |
| Cowlishaw et al. (2020) | Analytical cross-sectional | Inclusion criteria clearly defined | Unclear |
|  |  | Study subjects and setting described | Yes |
|  |  | Exposure measured validly/reliably | Unclear |
|  |  | Objective, standard outcome criteria used | Yes |
|  |  | Confounding factors identified | Yes |
|  |  | Strategies to deal with confounding | Unclear |
|  |  | Outcomes measured validly/reliably | Yes |
|  |  | Appropriate statistical analysis | Yes |
|  |  | Overall risk of bias | Moderate |
| Matthews et al. (2019) | Analytical cross-sectional | Inclusion criteria clearly defined | Yes |
|  |  | Study subjects and setting described | Yes |
|  |  | Exposure measured validly/reliably | Unclear |
|  |  | Objective, standard outcome criteria used | Yes |
|  |  | Confounding factors identified | Yes |
|  |  | Strategies to deal with confounding | Yes |
|  |  | Outcomes measured validly/reliably | Yes |
|  |  | Appropriate statistical analysis | Yes |
|  |  | Overall risk of bias | Moderate |
| Reifels et al. (2018) | Analytical cross-sectional | Inclusion criteria clearly defined | Yes |
|  |  | Study subjects and setting described | Yes |
|  |  | Exposure measured validly/reliably | Unclear |
|  |  | Objective, standard outcome criteria used | Yes |
|  |  | Confounding factors identified | Yes |
|  |  | Strategies to deal with confounding | Yes |
|  |  | Outcomes measured validly/reliably | Yes |
|  |  | Appropriate statistical analysis | Yes |
|  |  | Overall risk of bias | Low |
| Edwards et al. (2024) | Cohort | Two groups similar/recruited from same population | Yes |
|  |  | Exposures measured similarly | Yes |
|  |  | Exposure measured validly/reliably | Yes |
|  |  | Confounders identified | Yes |
|  |  | Confounders managed | Yes |
|  |  | Participants free of outcome at start | Unclear |
|  |  | Outcomes measured validly/reliably | Yes |
|  |  | Follow-up time sufficient | Yes |
|  |  | Follow-up complete / described | Yes |
|  |  | Loss to follow-up addressed | Yes |
|  |  | Stats appropriate | Yes |
|  |  | Overall risk of bias | Low |
| Dey et al. | Quasi-Experimental Studies | Cause/effect clear (exposure precedes outcome) | Yes |
|  |  | Participants similar in comparisons | NA |
|  |  | Participants treated similarly other than exposure | Unclear |
|  |  | Control group present | NA |
|  |  | Multiple measurements pre/post | Yes |
|  |  | Complete follow-up | Yes |
|  |  | Outcomes measured consistently/reliably | Yes |
|  |  | Outcomes measured similarly | Yes |
|  |  | Appropriate analysis | Yes |
|  |  | Overall risk of bias | Moderate |
| Hanigan et al. | Quasi-Experimental Studies | Cause/effect clear (exposure precedes outcome) | Yes |
|  |  | Participants similar in comparisons | NA |
|  |  | Participants treated similarly other than exposure | Unclear |
|  |  | Control group present | NA |
|  |  | Multiple measurements pre/post | Yes |
|  |  | Complete follow-up | Yes |
|  |  | Outcomes measured consistently/reliably | Yes |
|  |  | Outcomes measured similarly | Yes |
|  |  | Appropriate analysis | Yes |
|  |  | Overall risk of bias | Moderate |
| Xu et al | Quasi-Experimental Studies | Cause/effect clear (exposure precedes outcome) | Yes |
|  |  | Participants similar in comparisons | NA |
|  |  | Participants treated similarly other than exposure | Unclear |
|  |  | Control group present | NA |
|  |  | Multiple measurements pre/post | Yes |
|  |  | Complete follow-up | Yes |
|  |  | Outcomes measured consistently/reliably | Yes |
|  |  | Outcomes measured similarly | Yes |
|  |  | Appropriate analysis | Yes |
|  |  | Overall risk of bias | Moderate |
| De Leo et al. | Quasi-Experimental Studies | Cause/effect clear (exposure precedes outcome) | Yes |
|  |  | Participants similar in comparisons | Unclear |
|  |  | Participants treated similarly other than exposure | Unclear |
|  |  | Control group present | NA |
|  |  | Multiple measurements pre/post | Yes |
|  |  | Complete follow-up | Yes |
|  |  | Outcomes measured consistently/reliably | Yes |
|  |  | Outcomes measured similarly | Yes |
|  |  | Appropriate analysis | Yes |
|  |  | Overall risk of bias | Moderate |
| Guiney et al. (2012) | Case series | Clear inclusion criteria | Yes |
|  |  | Condition measured standardly | Yes |
|  |  | Valid methods for identification | Yes |
|  |  | Consecutive inclusion | Unclear |
|  |  | Demographics clearly reported | Yes |
|  |  | Clinical info clearly reported | Yes |
|  |  | Outcomes/follow-up clearly reported | NA |
|  |  | Site/clinic demographic info | NA |
|  |  | Statistical analysis appropriate | Yes |
|  |  | Overall risk of bias | Moderate |
| Kunde et al. (2019) | Case series | Clear inclusion criteria | Yes |
|  |  | Condition measured standardly | Yes |
|  |  | Valid methods for identification | Yes |
|  |  | Consecutive inclusion | Unclear |
|  |  | Demographics clearly reported | No |
|  |  | Clinical info clearly reported | Yes |
|  |  | Outcomes/follow-up clearly reported | NA |
|  |  | Site/clinic demographic info | NA |
|  |  | Statistical analysis appropriate | Yes |
|  |  | Overall risk of bias | Moderate |
| du Toit et al. (2026) | Cohort | Two groups similar/recruited from same population | Yes |
|  |  | Exposures measured similarly | Yes |
|  |  | Exposure measured validly/reliably | Yes |
|  |  | Confounders identified | Yes |
|  |  | Confounders managed | Unclear |
|  |  | Participants free of outcome at start | Yes |
|  |  | Outcomes measured validly/reliably | Yes |
|  |  | Follow-up time sufficient | Yes |
|  |  | Follow-up complete / described | Yes |
|  |  | Loss to follow-up addressed | Yes |
|  |  | Stats appropriate | Yes |
|  |  | Overall risk of bias | Low |
